# C-Reactive Protein-to-Serum Chloride Ratio: A Novel Marker of All-Cause Mortality in Maintenance Haemodialysis Patients

**DOI:** 10.3390/medicina60111765

**Published:** 2024-10-28

**Authors:** Francisco Valga, Tania Monzón, José C. De la Flor, Angelo Santana-del-Pino, Nicanor Vega-Díaz, Ana Yurena Sanchez-Santana, Gloria Antón-Pérez, Sergio Ruiz-Santana, José C. Rodríguez-Pérez, Patricia Perez-Borges

**Affiliations:** 1 Department of Nephrology, Doctor Negrin University Hospital of Gran Canaria, 35010 Las Palmas de Gran Canaria, Spain; evalamae@gobiernodecanarias.org (F.V.); tmonvaz@gobiernodecanarias.org (T.M.); aysansane@gobiernodecnarias.org (A.Y.S.-S.); pperbor@gobiernodecanarias.org (P.P.-B.); 2 Biomedicine Research Program, Doctoral School, University of Las Palmas de Gran Canaria, 35001 Las Palmas de Gran Canaria, Spain; nicanorjesus.vega@ulpgc.es (N.V.-D.); sergio.ruiz@ulpgc.es (S.R.-S.); 3 Department of Nephrology, Hospital Central de la Defensa Gómez Ulla, 28047 Madrid, Spain; 4 Department of Medicine and Medical Specialties, Faculty of Medicine, Alcala University, 28805 Madrid, Spain; 5 Mathematics Department, University of Las Palmas de Gran Canaria, 35017 Las Palmas de Gran Canaria, Spain; angelo.santana@ulpgc.es; 6 Department of Nephrology, Avericum Hemodialysis Centers, 35214 Telde, Spain; gloria.anton@avericum.com; 7 Intensive Care Unit, Doctor Negrin University Hospital of Gran Canaria, 35010 Las Palmas de Gran Canaria, Spain; 8 Postgraduate School and Research, University Fernando Pessoa-Canarias, 35450 Santa María de Guia, Spain; jcrodriguez@ufpcanarias.es

**Keywords:** serum chloride, C-reactive protein, haemodialysis, chronic kidney disease, bioimpedance, mortality

## Abstract

*Background and Objectives*: hypochloremia is an emerging risk factor for mortality in patients with chronic kidney disease. The pathophysiological mechanisms of this finding are not very clear. Some studies suggest the influence of inflammation as a synergistic factor, so we set out to analyse the association of a novel C-reactive protein-to-serum chloride ratio (CRP/Cl^−^) with the prognosis of maintenance haemodialysis patients and to assess its relationship with fluid status and body composition measured by bioimpedance. *Materials and Methods*: the present work is a retrospective cohort study of maintenance haemodialysis patients from our chronic outpatient haemodialysis programme between 1 January 2022 and 31 December 2022. (*n* = 281). Survival time was collected for all patients and analysed using the Kaplan–Meier method. A Cox proportional hazards regression model was used to evaluate survival probabilities. Variables included in the model were selected using a stepwise selection procedure based on the corrected Akaike information criterion (AICc), which balances model fit and complexity. *Results*: during a median follow-up of 306 days, 34 patients died. Patients in the fourth quartile of the CRP/Cl^−^ (>0.118 mg/mEq) had higher overall mortality (log-rank test, *p* = 0.0011). In the Cox multivariate analysis, the variables significantly associated with higher mortality were higher modified Charlson index (MCI), lower body surface area (BSA), lower interdialytic weight gain (IDWG), and higher CRP/Cl^−^ ratio. The latter variable was independently associated with higher overall mortality (adjusted hazard ratio = 1.027; 95% confidence interval [CI], 1.000–1.055 *p* = 0.0469). *Conclusions*: Higher CRP/Cl^−^ ratio values were associated with higher all-cause mortality in our maintenance haemodialysis patients.

## 1. Introduction

Recently, serum chloride (Cl^−^) has been gaining relevance as a prognostic marker in different cohorts of patients suffering from chronic kidney disease (CKD) [1,2,3,4,5]. Previously, it has been established as an excellent marker of mortality under other conditions such as hypertension [6], heart failure [7,8], and sepsis [9]. The underlying mechanisms remain unclear [5].

Some studies have reported a greater significance of this ion compared to sodium, especially in relation to the prognosis in some patient cohorts [1].

With regard to heart failure, it is of particular interest to note that lower chloride values have been associated with higher renin levels and worse results of decongestive treatments, resulting in diuretic resistance [7,10,11].

One possible explanation lies in the key role played by Cl^−^ in tubuloglomerular feedback: it is the intratubular concentration of this anion that suppresses renin activity through a direct effect on the macula densa. Furthermore, from an experimental point of view, there is a significant interaction between with-no-lysine (WNK) located in the distal tubule Na^+^-Cl^−^ channels and Cl^−^ [1,11,12].

After observing that, in patients at high cardiovascular risk, hypochloremia exerts a predictive role for mortality risk, our group decided to extrapolate these results to patients on haemodialysis, establishing the relationship between hypochloremia and increased all-cause and cardiovascular mortality in incident patients on chronic haemodialysis [5].

Inflammation, in terms of C-reactive protein (CRP), was an important variable in our previous studies [5,13]. It is a recognized prognostic marker in haemodialysis patients [13,14], and there are in vitro data suggesting a possible interaction between Cl^−^ and inflammation [11,15]. From a clinical point of view, results from some groups also suggest the existence of this relationship, as CRP levels have been found to be significantly higher in the quartile with lower Cl^−^ levels [2].

On the other hand, bioimpedance analysis (BIA) has become increasingly relevant in the assessment of body composition due to its simplicity, cost, and efficiency [16]. There is growing evidence of its usefulness as a clinical tool in CKD patients [17]: for example, volume overload estimated by this technique has been shown to be an independent mortality risk factor in these patients [18].

Furthermore, inflammation exerts a significant influence on several key physiological processes, including energy balance, body composition, and functionality. A correlation has been identified between this parameter and the phase angle, whereby an increase in inflammatory markers is associated with a reduction in the phase angle observed in bioimpedance analysis [19].

We, therefore, propose that there may be a triangular relationship among inflammation, serum chloride, and body composition, producing a synergistic deleterious effect among them.

In comparison to our previous study, which was conducted on patients who had recently initiated haemodialysis, the present study analyses a different population: patients who are undergoing maintenance haemodialysis. These patients receive greater vintage levels as part of the treatment and have a lower probability of having residual diuresis.

Therefore, the principal aim of our study is to evaluate the relationship between a new ratio that would consider both inflammation and serum chloride values like the C-reactive protein-to-serum chloride ratio (CRP/Cl^−^) and mortality. As we suspect that Cl^−^ values might be affected by changes in the various body compartments [5], we analyse the relationship between this new index and the parameters obtained through bioimpedance.

## 2. Materials and Methods

### 2.1. Study Design and Population

A multicentre retrospective cohort study was designed to include 281 maintenance haemodialysis patients from our two outpatient centres between 1 January 2022 and 31 December 2022. The inclusion criteria were as follows: over 18 years of age, regular maintenance haemodialysis for ≥3 months, and the patience needed to have undergone at least one single frequency bioimpedance (BIA) assessment (BIA 101 AKERN^®^, Florence, Italy) at the end of their haemodialysis session.

### 2.2. Data Collection

The electronic medical records of the included patients (Nefrosoft^®^ (version 7.2.2) and Selene-Drago^®^) were reviewed. Baseline demographic variables were sex, age, type of vascular access, aetiology of renal disease, diuretic use, modified Charlson index (excluding age and renal disease) (MCI) [20], cause and date of death.

Anthropometric and haemodialysis adequacy variables were: haemodialysis vintage (days), technique (standard or online haemodiafiltration), height (cm), pre-haemodialysis weight (kg), post-haemodialysis weight (kg), body mass index (BMI) (kg/m^2^), interdialytic weight gain (IDWG) (kg), ultrafiltration per mL/kg/hour, session duration (minutes), membrane type, dialyser surface area (m^2^), Kt obtained by ionic dialysance (Kt) (L), body surface area (BSA) calculated using the DuBois formula (m^2^), total body water (V) (L) (obtained by using bioimpedance and according to the Watson and Hume–Weyers formulae), target Kt adjusted to body surface area according to Lowrie’s formula (KtBSA) (L), KtBSA-Kt = ΔKt (L), Kt/V (with V obtained by using bioimpedance, the Watson formula, or the Hume–Weyers formula).

Analytical blood variables were obtained before starting the haemodialysis session, prior to bioimpedance, and included sodium (Na^+^) (mEq/L), potassium (K^+^) (mEq/L), chloride (Cl^−^) (mEq/L), bicarbonate (HCO_3_^−^) (mEq/L), CRP (mg/L), albumin (g/dL), gap anion (mmol/L), and CRP/Cl^−^ ratio (mg/mEq). Hypochloremia was considered when Cl^−^ < 98 mEq/L. High CRP levels were considered when CRP > 4.30 mg/L and lower CRP levels were considered when CRP ≤ 4.30 mg/L.

Our BIA equipment recorded resistance (Rz) (Ω) and reactance (Xc) (Ω) values. These data were inserted in the BIA software (BODYGRAM^®^ Dashboard 3.0, provided by the manufacturer) in order to calculate other variables like fat-free mass (FFM) (kg), total body water (TBW) (L), hydration of fat-free body mass (TBW:FFM), extracellular water (ECW) (L), intracellular water (ICW) (L), ECW/ICW, active cell mass (BCM) (kg), fat mass (FM) (kg), phase angle (PhA) (°), Na:K exchangeable ratio (Nae:Ke), total muscle mass (MM) (kg), basal metabolic rate (BMR) (kcal), and skeletal muscle mass (SMM) (kg). BIA measurements were taken at the end of the haemodialysis session after a 20 min rest period in the supine decubitus position.

### 2.3. Haemodialysis Technique

Flexia or Formula monitors (Bellco/Medtronic, Mirandola, Italy) with automatic data synchronisation and download were used. Ultrapure water was used in all cases as per the guidelines of the Spanish Society of Nephrology [21]. Patients were subjected to standard high-flow haemodialysis treatments and online haemodiafiltration techniques for 4 h, three times per week. The membranes used were from highly permeable and biocompatible polyethersulfone with a surface area ≥ 1.7 m^2^ (Elisium 17H, 19H, 21H, Nipro Medical Corporation, Osaka, Japan). Chloride concentration in the dialysate was 108.3 mEq/L. The prescribed blood flow (Qb) was the maximum allowed by the vascular access (300–500 mL/min).

### 2.4. Outcomes

Our primary outcomes were all-cause and cardiovascular mortality at the end of the study period. Cardiovascular mortality was defined as death due to cardiac arrest, heart failure, myocardial infarction, cerebrovascular accidents, or peripheral vascular disease. Survival time was calculated from the date of the BIA assessment until death, renal transplantation, loss to follow-up, or termination of the study: 31 December 2022. Secondary outcomes evaluated in this study included the relationship between CRP/Cl^−^ quartiles and bioimpedance-related variables.

### 2.5. Statistical Analysis

Baseline characteristics of the sample were described as follows: frequency and percentage for categorical variables, mean and standard deviation (SD) for normal continuous variables, and median and quartiles for non-normal continuous variables. Normality was assessed with the Shapiro–Wilk test.

Patients were divided into quartiles based on the CRP/Cl^−^ index [quartile 1 (<0.019 mg/mEq, N = 70), quartile 2 (0.019–0.043 mg/mEq, N = 70), quartile 3 (0.043–0.118 mg/mEq, N = 71), and quartile 4 (>0.118 mg/mEq, N = 70)]. The statistical analysis was conducted using appropriate tests based on the distribution of the data. For the comparison of mean values of a variable between two groups according to the death status, the student’s *t*-test was employed when the variable followed a normal distribution. In cases where the variable did not exhibit normality, the Wilcoxon rank-sum test was used as a non-parametric alternative. Normality was assessed using the Shapiro–Wilk test. For categorical variables, group comparisons were performed using the chi-square test.

For variables measured across the four groups ordered by CRP/Cl^−^ index quartiles, the focus was on determining whether there was an increasing or decreasing trend in the mean values of each variable across the groups defined according to the CRP/CI^−^ quartiles. The potential presence of a trend in the mean values across the four groups was assessed by using the Cuzick’s test for trend in the case of continuous variables, and the Cochran–Armitage test in the case of dichotomous variables. To ascertain in which specific quartiles the trend was evident, we conducted a step-down test, comparing successive quartiles using the Wilcoxon test, the *t*-test, or the proportion test (depending on which was the most appropriate, and applying a Hochberg adjustment for multiple comparisons. A *p*-value lower than 0.05 was considered to be statistically significant in all cases.

Kaplan–Meier survival curves were used for the entire cohort and for groups defined by the quartiles of the CRP/Cl^−^ ratio. Survival curves were compared using the log-rank test. Univariate and multivariate Cox regression models were developed to assess the association between the CRP/Cl^−^ ratio and mortality (both overall and cardiovascular). In the multivariate model, variable selection began by fitting univariate Cox models to all variables. Those with a significance level of *p* < 0.10 were initially included in the multivariate model [22,23]

Subsequently, a stepwise selection process based on the corrected Akaike information criterion (AICc) was implemented. This approach is particularly suited to small sample sizes, as it balances model fit and complexity. This method ensures that the most relevant predictors are retained while minimizing the risk of overfitting. The proportional hazards assumption was tested for all covariates, with statistical significance set to *p* < 0.05.

The statistical analysis was performed using the program R, version 4.3.1 (2023) (R Foundation for Statistical Computing, Vienna, Austria, http://www.R-project.org/) and SPSS^®^ 23.0 (SPSS Inc.; Chicago, IL, USA).

## 3. Results

### 3.1. Baseline Characteristics

The median age of the patients was 70 years. Women accounted for 31.8% of the sample and diabetes, as a cause of kidney disease, was present in 28.9% of the sample.

The median CRP/Cl^−^ was 0.04 (q25–q75: 0.02–0.12), and patients were grouped into quartiles of the CRP/Cl^−^ ratio.

In Table 1, significant trends can be observed between higher CRP/Cl^−^ quartiles and both increased mortality rates and longer durations of haemodialysis. Additionally, at the biochemical level, albumin levels showed a decreasing trend across the quartiles. No significant trends were found for MCI, age, technique, or vascular access.

Only dialysis vintage, albumin, and percentage of deaths showed a significant trend. The values of these variables were compared across successive quartiles of the CRP/Cl^−^ ratio. For dialysis vintage, a significantly lower value was observed in the first quartile (*p* = 0.028), with no significant differences in the remaining quartiles.

On the other hand, albumin exhibited significant differences across successive quartiles (*p* = 0.041 for the comparison between Q1 and Q2, *p* = 0.033 for Q2 vs. Q3, and *p* = 0.01 for Q3 vs. Q4). Regarding the proportion of deaths, no differences were observed across quartiles 1 to 3 (*p* > 0.134); however, there was a significant increase in mortality in quartile 4 (*p* = 0.0027) compared to the previous quartiles.

When the sample was classified according to death status, patients who died had a higher median CRP/Cl^−^ ratio, MCI, and age, as well as a lower median serum albumin, BSA, IDWG and BMI. In addition, a lower percentage of them were on online hemodiafiltration (Table 2).

### 3.2. Haemodialysis Adequacy

The median Kt was 56 L (q25–q75: 52–62). The target Kt adjusted to body surface area (KtBSA) was 50.55 ± 4.09 L, while the bioimpedance-adjusted Kt/V was 1.44 (q25–q75: 1.25–1.65). The average difference between KtBSA and Kt (ΔKt) was 6.04 ± 7.85 L.

In Table 3, a significant increasing trend can be observed between the higher CRP/Cl^−^ quartiles, on one side, and the higher Kt and ΔKt values, on the other.

When comparing Kt values across the quartiles of the CRP/Cl^−^ ratio, no significant differences were found between successive quartiles (*p* > 0.158), although the difference between the first and fourth quartile was significant (*p* = 0.0028). The same result was obtained for ΔKt, with no significant differences between successive quartiles (*p* > 0.1477), although a significant difference between the first and last quartile was found (*p* = 0.013). This result indicates that both variables experienced a slow increase across quartiles, leading to a significant overall difference.

According to the death status, patients who died had a lower KtBSA (L) and a higher Kt/V adjusted by bioimpedance. In addition, they had a lower interdialytic gain. There were no significant differences in Kt or ΔKt between the two groups (Table S1).

### 3.3. Bioimpedance-Related Parameters

The median phase angle was 4.8° (q25–q75: 4.20–5.50). No significant increasing or decreasing trend was observed in any of these variables with the quartiles of the CRP/Cl^−^ ratio (Table S2).

According to the death status, patients who died had a lower phase angle, a higher Na:K exchangeable ratio (Nae:Ke), and lower intracellular water content, lean mass, and fat mass (Table 4). No differences in the hydration status (TBW:FFM) were observed when the sample was classified according to the death status 73.6% (73.3–75.9%) vs. 73.9% (73.3–78.7%) (alive vs. dead, respectively) (*p* = 0.207), although patients with mild overhydration were detected in both groups.

### 3.4. CRP/Cl^−^ Ratio and Mortality

During a median follow-up of 306 days (q25–q75: 240–326), 34 (12.1%) of the 281 patients died. The causes of death were: cardiovascular (*n* = 16, 47.1%), infectious (*n* = 12, 35.3%), and other (*n* = 6, 17.6%).

**Figure 1:**
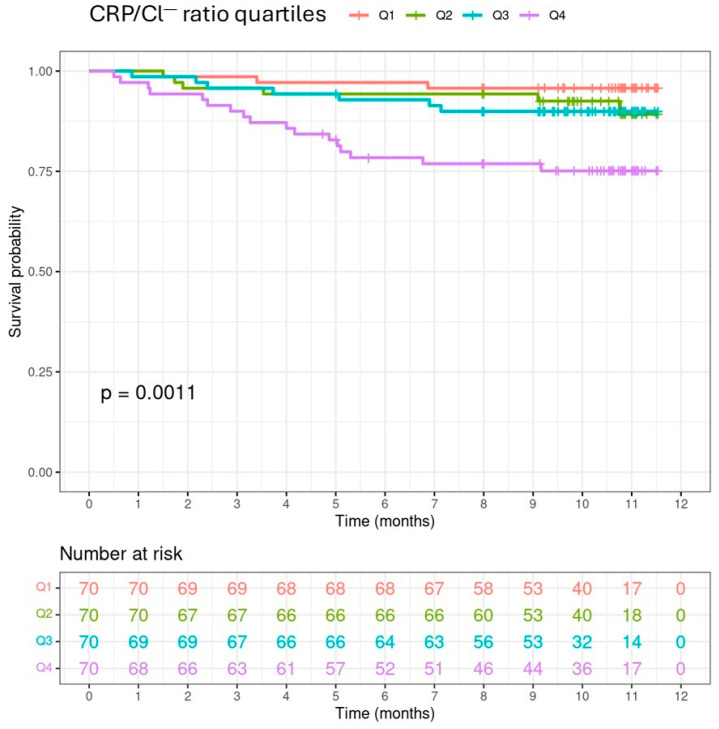
Kaplan–Meier estimate of overall survival probability. The *p*-value of the log-rank test (*p* = 0.0011) indicates significant differences in survival depending on the index. Post-hoc analyses for the log-rank test show that survival in the 4th quartile is significantly lower than that in the 1st (*p* = 0.0039), 2nd (*p* = 0.03242), and 3rd (*p* = 0.0458), being the differences between other pairs of curves not significant (*p* > 0.2851).

**Figure 2:**
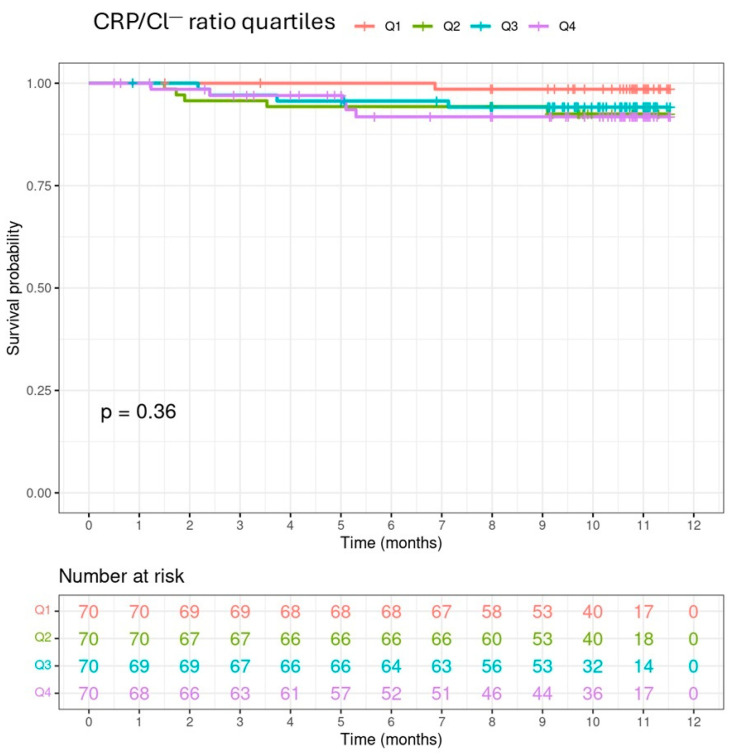
Kaplan–Meier estimate of cardiovascular survival probability.

The Kaplan–Meier survival analysis stratified by CRP/Cl^−^ ratio quartiles showed that patients belonging to the fourth quartile (>0.118 mg/mEq) experienced a higher overall mortality (log-rank test, *p* = 0.0011) (Figure 1), but this difference was not observed when cardiovascular mortality was analysed (log-rank test, *p* = 0.36) (Figure 2).

To identify, initially, which variables were significantly associated with survival, univariate Cox proportional hazard regression models were fitted to each one of the variables in the study. Only those variables for which the regression coefficient had a *p*-value < 0.10 are shown (Table 5).

These variables were included in a multivariate model. A stepwise selection procedure, guided by the corrected Akaike information criterion (AICc), which is well-suited to small sample sizes, was used to identify the most relevant variables. The variables ultimately selected were CRP/Cl^−^, albumin, MCI, BSA, IDWG, ΔKt, and ultrafiltration rate.

After adjusting for the remaining variables, the coefficient of CRP/Cl^−^ was positive (0.027, with se = 0.014), meaning that higher baseline values of CRP/Cl^−^ ratio were associated with lower survival (HR 1.027; 95% CI 1.000–1.055; *p* = 0.0469).

Furthermore, higher values of MCI (HR 1.165; 95% CI, 1.045–1.300; *p* = 0.006) and lower values of both BSA (HR 0.096; 95% CI 0.013–0.693) and IDWG (HR 0.481; 95% CI 0.243–0.955) were also statistically significantly associated with lower survival (Table 6).

## 4. Discussion

To the best of our knowledge, this is the first study on maintenance haemodialysis patients to demonstrate a significant and independent association between higher baseline CRP/Cl^−^ ratio values and increased all-cause mortality.

Cl^−^ has gained importance as a prognostic marker in different patient groups, such as patients suffering from heart failure, arterial hypertension, sepsis, and chronic kidney disease [3,4,6,7,8,9], and its relationship with inflammation has been documented by some in vitro studies [11,15,24].

A recent work by our group has shown that incident chronic haemodialysis patients in the groups with higher CRP and lower Cl^−^ values experienced higher mortality (*p* = 0.00044). Moreover, among the patients with lower CRP values, those with lower Cl^−^ values also experienced a higher mortality (*p* = 0.00044) [5], findings that overlap with those of our present study (Figure S1).

On the other hand, in this same study, patients belonging to the lowest Cl^−^ quartile group experienced a higher interdialytic gain and a higher overall and cardiovascular mortality, so we decided to analyse variables related to body composition.

In our study, no significant increasing or decreasing trends were observed in any of the variables obtained from bioimpedance when classified by quartile of the CPR/Cl^−^ ratio. This suggests that variations in chloride would not be related to changes in body composition, something which is consistent with the absence of significant trends in interdialytic weight gain as well.

However, when the sample was sub-grouped according to the death status, those who died had, among other variables, a lower TBW, ICW, BCM, FM, PhA, MM, BMR, and SMM. This indicates that patients who died were probably in worse nutritional conditions than the rest of the patients [17,25]. This is supported by a higher median Nae:Ke found in patients who died. It should be noted that a Nae:Ke value ≥ 1.22 implies a high risk of malnutrition [26].

A lower TBW is likely to have influenced a higher median Kt/V in patients who died, especially when Kt was similar between the two groups, a finding similar to those described by Perez-Garcia et al. [27]. However, none of these variables were associated with increased mortality in our adjusted Cox regression model.

A low phase angle may serve as an indicator of the breakdown of selective permeability in cell membranes or as an indicator of cell death. Caravaca et al. described in a study of patients with advanced chronic kidney disease without dialysis that those with a phase angle < 5.3° experienced higher mortality, a finding which is consistent with our results [28]. However, phase angle was not selected for the final model, probably due to the presence of other closely related nutritional variables such as albumin. To clarify this effect, a model was run forcing the inclusion of phase angle (Table S3): as it can be seen, this variable was not statistically significant and, as expected, albumin, BSA, and ΔKT disappeared in the final adjusted model. CRP/Cl^−^ ratio remained significant in this model.

In a study on haemodialysis patients (*n* = 92), Keber et al. [18] demonstrated a higher mortality in patients with a volume overload greater than 2.5 litres. However, the median IDWG in our sample was 2.10 litres, so they were not comparable. This, together with the fact that those who died had a similar ECW to those in the other group, leads us to conclude that our sample was very homogeneous in terms of volume overload.

It has recently been described that low serum chloride levels stimulate with-no-lysine protein kinases (WNK), a function that seems to be relevant in the macrophage response in sepsis [24]. This situation leads to increased synthesis of chloride and sodium transporters such as Na^+^-Cl^−^ cotransporter (NCC) or Na^+^-K^+^-2Cl^−^ (NKCC) and to increased activity of the renin–angiotensin–aldosterone axis [5,11,12].

Also, in some experimental studies, the tumor necrosis factor (TNF)-α has been found to increase the activity of some kinases, such as WKN1, so there may be a synergistic effect between hypochloremia and inflammation [11,15].

The main strength of our study is that it is the first observational study to analyse the relationship between the CRP/Cl^−^ ratio and overall mortality in maintenance haemodialysis patients as well as the use of BIA.

Some of the limitations of our study include its retrospective design, the short duration of the follow-up, and its circumscription to a single geographical area. We were unable to analyse patient comorbidity (especially congestive heart failure and cirrhosis). Neither baseline echocardiography nor residual diuresis data could be retrieved, and bioimpedance data were obtained only at the end of the haemodialysis session because of standard clinical practice.

## 5. Conclusions

Higher CRP/Cl^−^ ratio values are associated with higher all-cause mortality in our maintenance haemodialysis patient cohort. Prospective studies on this ratio are needed to clinically validate its relevance against other established markers.

## Figures and Tables

**Table 1:** Baseline characteristics of patients according to the quartiles of C-Reactive protein-to-serum chloride ratio.

Characteristics	Total N = 281	Q1 N = 70	Q2 N = 70	Q3 N = 71	Q4 N = 70	*p*-Value
Female, *n* (%)	89 (31.8)	22 (31.4)	22 (31.4)	27 (38.6)	18 (25.7)	0.4517
Age (years)	70 (59–77)	70 (60–76)	73 (62–78)	68 (58–76)	70 (58–77)	0.4616
Diabetic nephropathy, *n* (%)	81 (28.9)	24 (34.3)	17 (24.3)	18 (25.7)	22 (31.4)	0.5363
Death, *n* (%)	33 (11.8)	3 (4.3)	6 (8.6)	7 (10.0)	17 (24.3)	0.0033
Cause of death (cardiovascular), *n* (%)	5 (45.5)	1 (33.3)	5 (83.3)	4 (57.1)	5 (29.4)	0.1668
Technique (OL-HDF), *n* (%)	119 (42.5)	33 (47.1)	28 (40.0)	31 (44.3)	27 (38.6)	0.7351
Vascular access (AVF), *n* (%)	65 (58.9)	39 (55.7)	38 (54.3)	45 (64.3)	43 (61.4)	0.5965
MCI	9.00 (6.75–11.00)	9.00 (6.00–11.00)	9.00 (8.00–11.00)	9.00 (5.50–11.00)	9.00 (6.00–12.00)	0.2075
Dialysis vintage (days)	1135 (652–1863)	846 (578–1370)	1156 (568–1901)	1424 (861–2111)	1282 (702–2246)	0.0114
Diuretics (yes), *n* (%)	124 (44.3)	33 (47.1)	27 (38.6)	32 (45.7)	32 (45.7)	0.7429
BMI (kg/m^2^)	25.90 (22.75–30.13)	25.90 (22.35–29.30)	25.75 (22.65–30.10)	26.15 (22.68–30.73)	26.00 (23.50–29.45)	0.9035
BSA (m^2^)	1.85 (1.68–2.00)	1.83 (1.66–1.99)	1.79 (1.67–1.93)	1.87 (1.72–2.03)	1.87 (1.72–2.02)	0.2874
Sodium (mEq/L)	138 (136–140)	139 (137–140)	138 (137–141)	138 (136–140)	138 (136–139)	0.0857
Potassium (mEq/L), mean ± SD	5.01 ± 0.87	5.24 ± 0.85	4.89 ± 0.90	4.92 ± 0.81	5.00 ± 0.89	0.1353
Anion Gap (mmol/L), mean ± SD	22.84 ± 3.50	22.47 ± 3.48	22.73 ± 4.03	23.22 ± 2.85	22.95 ± 3.60	0.3040
HCO_3_^−^ (mEq/L)	20.35 (18.80–21.80)	20.45 (19.30–22.00)	20.70 (18.83–21.98)	20.10 (18.42–21.20)	20.35 (18.80–21.58)	0.5882
Albumin (g/dL)	3.90 (3.60–4.10)	4.00 (3.82–4.20)	3.90 (3.70–4.20)	3.80 (3.62–4.00)	3.70 (3.40–3.98)	<0.0001

The values are expressed as median (q25–q75) unless otherwise specified. The trend test *p*-value is shown. OL-HDF: online haemodiafiltration; AVF: arteriovenous fistula; MCI: modified Charlson index; BMI: body mass index; BSA: body surface area.

**Table 2:** Baseline characteristics according to death status.

Characteristics	Total N = 281	NO N = 247	YES N = 34	*p*-Value
Female, *n* (%)	89 (31.7)	77 (31.2)	12 (35.3)	0.6947
Age (years)	70 (59–77)	70 (58–76)	77 (71–81)	0.0006
MCI	9(7–11)	9(6–11)	11(9–12)	0.0004
Diabetic nephropathy, *n* (%)	82 (29.2)	70 (28.3)	12 (35.3)	0.4226
Technique (OL-HDF), *n* (%)	120 (42.7)	113 (45.7)	7 (20.6)	0.0055
Vascular access (AVF), *n* (%)	166 (59.1)	148 (59.9)	18 (52.9)	0.4613
Dialysis vintage (days)	1135 (652–1864)	1136 (652–1869)	1088 (669–1799)	0.5204
Diuretics (yes), *n* (%)	124 (44.1)	107 (43.3)	17 (50.0)	0.4679
BMI (kg/m^2^)	26(22.8–30.2)	26.3 (22.9–30.6)	24(21.65–26.95)	0.011
CRP/Cl^−^ ratio	0.04 (0.02–0.12)	0.04 (0.02–0.10)	0.12 (0.04–0.19)	0.0005
BSA (m^2^)	1.85 (1.68–2)	1.86 (1.72–2.02)	1.75 (1.59–1.87)	0.0036
IDWG (L)	2.10 (1.37–2.8)	2.20 (1.5–2.9)	1.65 (0.72–2.32)	0.0029
Hypochloremia, *n* (%)	72(25.6)	60 (24.3)	12 (35.3)	0.2083
High CRP, *n* (%)	139(49.5)	114 (46.2)	25 (73.5)	0.0032
Potassium (mEq/L), mean ± SD	5.01 ± 0.87	5.03 ± 0.88	4.84 ± 0.73	0.1564
Anion gap (mmol/L), mean ± SD	22.84 ± 3.50	22.91 ± 3.47	22.36 ± 3.70	0.4240
Sodium (mEq/L)	138 (136–140)	138 (137–140)	138 (135–139)	0.4770
HCO_3_^−^ (mEq/L)	20.40 (18.80–21.80)	20.40 (18.80–21.75)	20.30 (19.40–21.90)	0.5705
Albumin (g/dL)	3.90 (3.60–4.10)	3.90 (3.70–4.10)	3.70 (3.32–3.80)	<0.0001

The values are expressed as median (q25–q75) unless otherwise specified. The *p*-value to assess differences between groups is shown. OL-HDF: online haemodiafiltration; AVF: arteriovenous fistula; MCI: modified Charlson index; BMI: body mass index; BSA: body surface area; BMI: body mass index; CRP/Cl^−^: C-reactive protein-to-serum chloride ratio; IDWG: interdialytic weight gain.

**Table 3:** Baseline characteristics related to the adequacy of haemodialysis according to the quartiles of C-reactive protein-to-serum chloride ratio.

Characteristics	Total N = 281	Q1 N = 70	Q2 N = 70	Q3 N = 71	Q4 N = 70	*p*-Value
Kt (L)	56 (52; 62)	54 (50; 60)	56 (51; 61)	56 (52; 62)	58 (54; 64)	0.0405
KtBSA (L), mean ± SD	50.55 ± 4.09	50.18 ± 4.13	49.99 ± 3.93	50.96 ± 3.65	51.09 ± 4.57	0.0912
ΔKt (L), mean ± SD	6.04 ± 7.85	4.77 ± 8.38	5.94 ± 8.37	5.78 ± 6.78	7.66 ± 7.66	0.0424
Ultrafiltration rate (mL/kg/h)	8.20 (6.06–10.45)	8.57 (5.88–11.06)	8.51 (6.15–10.74)	7.97 (6.40–9.85)	7.76 (5.41–10.19)	0.5375
Negative fluid balance per session (L)	2.30 (1.50–2.90)	2.30 (1.23–2.80)	2.20 (1.60–2.90)	2.25 (1.50–2.90)	2.35 (1.33–2.98)	0.9866
Kt/V (BIA)	1.44 (1.25–1.64)	1.40 (1.19–1.57)	1.48 (1.28–1.72)	1.44 (1.25–1.60)	1.47 (1.28–1.66)	0.2374
Kt/V (W)	1.49 (1.31–1.69)	1.46 (1.29–1.65)	1.53 (1.30–1.73)	1.48 (1.32–1.67)	1.48 (1.34–1.69)	0.7661
Kt/V (HW)	1.44 (1.26–1.62)	1.43 (1.24–1.62)	1.47 (1.24–1.66)	1.42 (1.28–1.57)	1.45 (1.29–1.64)	0.8613
IDWG (L)	2.10 (1.35–2.80)	2.20 (1.30–3.00)	2.00 (1.50–2.60)	2.20 (1.33–3.00)	2.10 (1.30–2.70)	0.7103

The values are expressed as median (q25–q75) unless otherwise specified. The trend test *p*-value is shown. ΔKt = target Kt − Kt; KtBSA: target Kt adjusted to body surface area according to Lowrie’s formula; Kt/V (BIA): Kt/V obtained by bioimpedance; Kt/V (W): Kt/V obtained by using the Watson’s formula; Kt/V (HW): Kt/V obtained by using the Hume–Weyer’s formula; IDWG: interdialytic weight gain.

**Table 4:** Variables related to bioimpedance according to the death status.

Characteristics	Total N = 281	NO N = 247	YES N = 34	*p*-Value
PhA (°)	4.80 (4.20–5.50)	4.90 (4.30–5.60)	4.15 (3.70–5.07)	0.0014
Xc (Ω)	44 (36–54)	45 (37–54)	38 (33–53)	0.1398
Rz (Ω), mean ± SD	535.15 ± 100.20	532.33 ± 98.51	555.66 ± 111.14	0.2517
Nae:Ke	1.13 (1.00–1.39)	1.11 (0.99–1.36)	1.32 (1.05–1.67)	0.0043
FFM (kg)	52 (45; 59)	52 (46; 59)	46 (42; 55)	0.0154
ECW (L)	20.20 (17.60–22.70)	20.30 (17.60–22.75)	19.55 (17.45–22.55)	0.5991
ICW (L)	18.80 (15.40–22.20)	19.10 (15.80–22.40)	16.60 (13.07–19.43)	0.0027
TBW (L)	39 (34–44)	39 (35–44)	35 (31–42)	0.0223
TBW (W) (L)	38 (33–43)	38 (34–43)	34 (30–39)	0.0045
TBW (HW) (L)	40 (34–44)	40 (35–44)	38 (29–42)	0.0215
ECW/ICW	1.08 (0.93–1.27)	1.05 (0.91–1.23)	1.27 (1.02–1.46)	0.0016
BCM (kg)	24.60 (20.10–29.40)	24.90 (20.60–29.95)	21.90 (16.97–25.52)	0.0014
FM (kg)	21.40 (15.00–28.50)	22.40 (15.20–29.55)	17.55 (12.25–25.75)	0.0222
MM (kg)	31 (26–36)	32 (26–37)	27.75 (21.88–32.30)	0.0021
BMR (kcal)	1462 (1334–1604)	1471 (1348–1618)	1386 (1241–1490)	0.0014
SMM (kg)	24.50 (20.20–28.40)	25.10 (20.65–28.70)	22.30 (18.62–27.17)	0.0441

The values are expressed as median (q25–q75) unless otherwise specified. The *p*-value to assess differences between groups is shown (using either the student’s *t*-test or the Wilcoxon test, depending on which test was more appropriate). PhA: phase angle; Rz: resistance; Xc: reactance; FFM: fat-free mass; TBW: total body water obtained by bioimpedance; TBW (W): total body water obtained through Watson’s formula; TBW (HW): total body water obtained through Hume–Weyers’ formula. ECW: extracellular water; ICW: intracellular water; ECW/ICW: extracellular to intracellular water ratio; BCM: active cell mass; FM: fat mass; Nae:Ke: Na:K exchangeable ratio; MM: total muscle mass; BMR: basal metabolic rate; SMM: skeletal muscle mass.

**Table 5:** Cox proportional hazard regression model (unadjusted Cox analysis).

Covariates	Beta (se)	HR	HR CI 95%	*p*-Value
CRP/Cl^−^	0.033 (0.012)	1.033	[1.009, 1.058]	0.0068
MCI	0.190 (0.051)	1.209	[1.095, 1.336]	0.0066
Albumin	−1.465 (0.374)	0.231	[0.111, 0.481]	<0.0001
CRP	0.033 (1.033)	1.033	[1.009, 1.058]	0.0066
Age	0.046 (0.017)	1.047	[1.013, 1.083]	0.0068
FFM	−0.052 (0.020)	0.949	[0.912, 0.988]	0.0101
TBW	−0.055 (0.025)	0.947	[0.901, 0.995]	0.0318
ICW	−0.124 (0.041)	0.883	[0.815, 0.957]	0.0023
ECW/ICW	1.389 (0.459)	4.010	[1.631, 9.858]	0.0025
BCM	−0.094 (0.029)	0.910	[0.860, 0.964]	0.0013
FM	−0.036 (0.017)	0.964	[0.934, 0.996]	0.0283
PhA	−0.559 (0.186)	0.572	[0.397, 0.822]	0.0026
Nae:Ke	0.886 (0.360)	2.424	[1.197, 4.911]	0.0139
MM	−0.079 (0.025)	0.924	[0.879, 0.971]	0.0018
BMR	−0.003 (0.001)	0.997	[0.995, 0.999]	0.0013
BMI	−0.075 (0.034)	0.928	[0.869, 0.991]	0.0264
SMM	−0.054 (0.030)	0.947	[0.894, 1.004]	0.0668
Negative fluid balance per session	−0.342 (0.162)	0.710	[0.517, 0.976]	0.0347
Ultrafiltration rate	−0.001 (0.001)	0.999	[0.998, 1.000]	0.0644
BSA	−2.478 (0.833)	0.084	[0.016, 0.429]	0.0029
TBW (W)	−0.080 (0.028)	0.923	[0.875, 0.975]	0.0039
TBW (HW)	−0.066 (0.026)	0.936	[0.890, 0.985]	0.0113
KtBSA	−0.133 (0.044)	0.876	[0.803, 0.955]	0.0028
ΔKt	0.035 (0.021)	1.036	[0.994, 1.080]	0.0964
Kt/V (BIA)	1.405 (0.568)	4.076	[1.339, 12.409]	0.0134
Kt/V (W)	1.668 (0.538)	5.299	[1.846, 15.213]	0.0019
Kt/V (WH)	1.502 (0.556)	4.492	[1.509, 13.367]	0.0069
IDWG	−0.431 (0.156)	0.650	[0.479, 0.882]	0.0057
Gender	0.744 (0.420)	2.104	[0.924, 4.790]	0.0766
Vascular access	−0.849 (0.396)	0.428	[0.197, 0.930]	0.0320

HR: hazard ratio; CRP/Cl^−^: C-reactive protein-to-serum chloride ratio; CRP: C-reactive protein; MCI: modified Charlson index; PhA: phase angle; FFM: fat-free mass; TBW: total body water obtained by bioimpedance; TBW (W): total body water obtained by using Watson’s formula; TBW (HW): total body water obtained by using Hume–Weyers’ formula. ECW: extracellular water; ICW: intracellular water; ECW/ICW: extracellular to intracellular water ratio; BCM: active cell mass; FM: fat mass; Nae:Ke: Na:K exchangeable ratio; MM: total muscle mass; BMR: basal metabolic rate; SMM: skeletal muscle mass; BMI: body mass index; BSA: body surface area; ΔKt = KtBSA − Kt; KtBSA: target Kt adjusted to body surface area according to Lowrie’s formula; Kt/V (BIA): Kt/V obtained by bioimpedance; Kt/V (W): Kt/V obtained by using Watson’s formula; Kt/V (HW): Kt/V obtained by using Hume–Weyer’s formula; IDWG: interdialytic weight gain.

**Table 6:** Multivariate Cox regression model with automatic variable selection using the AICc criterion.

Covariate	Beta (se)	HR	HR CI95%	*p*-Value
Albumin	−0.720 (0.402)	0.487	[0.222; 1.070]	0.0733
MCI	0.153 (0.056)	1.165	[1.045; 1.300]	0.0060
BSA	−2.341 (1.008)	0.096	[0.013; 0.693]	0.0202
CRP/Cl^−^	0.027 (0.014)	1.027	[1.000; 1.055]	0.0469
IDWG	−0.731 (0.349)	0.481	[0.243; 0.955]	0.0363
ΔKt	0.037 (0.025)	1.038	[0.989; 1.090]	0.1335
Ultrafiltration rate (mL/kg/h)	0.002 (0.001)	1.002	[0.999; 1.004]	0.1498

HR: hazard ratio; CRP/Cl^−^: C-reactive protein-to-serum chloride ratio; MCI: modified Charlson index; BSA: body surface area; IDWG: interdialytic weight gain; ΔKt = KtBSA − Kt.

## Data Availability

No new data were created or analysed in this study. The data used to support the findings of this study are available from the corresponding author upon request (contact F.V., evalamae@gobiernodecanarias.org). We confirm that all figures and tables are the original work of this manuscript’s authors. All have been created by the authors of this manuscript, they have not been adapted from the work of other authors, and do not present an online link.

## Supplementary Materials

The following supporting information can be downloaded at: https://www.mdpi.com/article/10.3390/medicina60111765/s1, Table S1: Variables related to the adequacy of haemodialysis according to the death status; Table S2: Characteristics related to bioimpendance according to the quartiles of the C-reactive protein-to-serum chloride ratio; Table S3: Multivariate Cox regression model forcing the inclusion of phase angle; Figure S1: Kaplan–Meier estimate of all-cause survival probability by CRP and serum chloride levels.
