# Supplementary material for: C-Reactive Protein-to-Serum Chloride Ratio: A Novel Marker of All-Cause Mortality in Maintenance Haemodialysis Patients
Source: Medicina (Kaunas). 2024 Oct 28;60(11):1765. doi: 10.3390/medicina60111765 (PMC11596270; doi:10.3390/medicina60111765)
Supplement: Supplementary file 1 [file medicina-60-01765-s001.zip › medicina-3239219-supplementary.pdf]

Table S1. Variables related to adequacy of haemodialysis according to death status

Characteristics	Total<br>N=281	NO<br>N=247	SI<br>N=34	p-value
Kt (L)	56 (52-62)	56 (52- 62)	56 (53- 64)	0.7229
KtBSA (L), mean ± SD	50.56 ± 4.09	50.83 ± 4.10	48.62 ± 3.46	0.0013
ΔKt (L), mean ± SD	6.06 ± 7.84	5.77 ± 7.94	8.16 ± 6.84	0.0669
Kt/V (BIA)	1.44 (1.25-1.64)	1.43 (1.24-1.61)	1.58 (1.42-1.81)	0.0084
Kt/V (W)	1.49 (1.31-1.69)	1.47 (1.30-1.66)	1.68 (1.43-1.85)	0.0021
Kt/V (WH)	1.44 (1.26-1.62)	1.42 (1.25-1.59)	1.60 (1.36-1.74)	0.0077
Ultrafiltration rate (mL/kg/hour)	8.17 (6.00-10.43)	8.31 (6.13-10.37)	7.44 (5.37-10.74)	0.5744
IDWG	2.10 (1.37-2.80)	2.20 (1.50-2.90)	1.65 (0.72-2.32)	0.0029

The values are expressed as median (q25-q75) unless otherwise specified. Trend test p-value is shown. ΔKt=Target Kt- Kt; KtBSA: target Kt adjusted to body surface area according to Lowrie's formula; Kt/V (BIA): Kt/V obtained by bioimpedance; Kt/V (W): Kt/V obtained by Watson's formula; Kt/V (HW): Kt/V obtained by Hume-Weyer's formula; IDWG: interdialytic weight gain

Table S2. Characteristics related to bioimpendance according to quartile of C-reactive protein-to-serum chloride ratio.

**Characteristics**	**Total N=281**	**Q1 N=70**	**Q2 N=70**	**Q3 N=71**	**Q4 N=70**	**p-value**
PhA (°)	4.80 (4.20-5.50)	4.90 (4.23-5.50)	4.80 (4.30-5.68)	4.80 (4.10-5.38)	4.70 (3.92- 5.57)	0.4669
Xc (Ω)	44 (36-54)	45 (38-53)	47 (38-55)	43 (36-53)	43 (35-53)	0.5003
Rz (Ω), mean ± SD	535.24 ± 100.36	521.88 ± 96.57	545.21 ± 103.14	537.09 ± 103.89	536.78 ± 98.41	0.4964
Nae:Ke	1.13 (1.00-1.39)	1.16 (1.02-1.35)	1.08 (0.95-1.32)	1.15 (1.01-1.48)	1.15 (0.99- 1.46)	0.4986
FFM (kg)	52 (45-59)	54 (45-59)	50 (44-57)	52 (46-59)	52 (46-60)	0.3752
ECW (L)	20.20 (17.60-22.63)	20.45 (17.27-22.82)	19.40 (17.10-21.38)	20.50 (18.35-23.20)	20.60 (18.20-23.05)	0.0957
ICW (L)	18.80 (15.40-22.20)	19.30 (15.52-23.00)	17.95 (15.03-21.68)	19.10 (15.35-21.70)	18.40 (15.50-23.02)	0.5584
TBW (L)	39 (34-44)	40 (34-44)	37 (33-42)	39 (35-44)	40 (35- 45)	0.3316
TBW (W) (L)	38 (33-43)	38 (32-43)	37 (33-41)	37 (33-42)	40 (34- 43)	0.4005
TBW (HW) (L)	40 (34-44)	40 (32-44)	39 (33-42)	39 (36-43)	41 (35-45)	0.4183
ECW/ICW	1.07 (0.92-1.27)	1.05 (0.93-1.24)	1.08 (0.90-1.22)	1.07 (0.96-1.29)	1.12 (0.91-1.35)	0.4551
BCM (kg)	24.60 (20.10-29.42)	25.15 (20.52-30.85)	23.80 (19.40-29.08)	24.90 (19.85-28.40)	24.05 (20.75-30.08)	0.6425
FM (kg)	21.35 (15.00-28.42)	20.60 (13.85-25.98)	22.00 (14.93-27.62)	24.10 (15.22-31.02)	19.80 (15.53-29.50)	0.3374
MM (Kg)	31 (26-36)	32 (26-38)	29.55 (25.05-35.80)	32 (26-36)	30 (26-36)	0.6024
BMR (Kcal)	1462 (1334-1605)	1479 (1345-1645)	1440 (1313-1594)	1472 (1325-1574)	1448 (1351-1622)	0.6404
SMM (kg)	24.50 (20.17-28.40)	26.75 (20.02-29.55)	23.50 (19.68-27.03)	24.15 (20.50-28.78)	25.45 (21.30-28.35)	0.2190

The values are expressed as median (q25-q75) unless otherwise specified. Trend test p-value is shown. PhA: phase angle; Rz: resistance; Xc: reactance; FFM: fat-free mass; TBW: total body water obtained by bioimpedance; TBW (W): total body water obtained by Watson's formulae; TBW (HW): total body water obtained by Hume-Weyers' formula. ECW: extracellular water; ICW: intracellular water; ECW/ICW; Extracellular to intracellular water ratio; BCM: active cell mass; FM: fat mass; Nae:Ke: Na:K exchangeable ratio); MM: total muscle mass; BMR: basal metabolic rate and SMM: skeletal muscle mass.

Table S3. Multivariate Cox regression model forcing the inclusion of Phase Angle[a]

Covariate	beta(se)	HR	HR CI95%	P
PhA	-0.281 (0.204)	0.755	[0.506; 1.127]	0.1695
MCI	0.167 (0.056)	1.181	[1.059; 1.318]	0.0029
FM	-0.034 (0.020)	0.966	[0.929; 1.005]	0.0882
CRP/Cl⁻	0.030 (0.013)	1.031	[1.005; 1.057]	0.0167
IDWG	-0.755 (0.339)	0.470	[0.242; 0.913]	0.0259
Kt/V (HW)	1.122 (0.685)	3.071	[0.801; 11.769]	0.1017
Ultrafiltration rate	0.002 (0.001)	1.002	[0.999; 1.005]	0.1332

[a]In this model the variables MCI, CRP/Cl⁻ ratio, IDWG and ultrafiltration rate are kept. Albumin, BSA and ΔKt have disappeared. The effect that in the previous model (Table 6) was captured by the variables ΔKt and BSA is probably captured in the new model by the variables Kt/V (HW) and FM. It is important to note that ΔKt and Kt/V (HW) are significantly associated (pearson correlation r=0.743) (variables related to haemodialysis adequacy) and something similar happens with BSA and FM (correlation r=0.75) (variables related to body composition). The disappearance of albumin can perhaps be explained by the presence of PhA as these are variables related to nutrition. In any case, the AICc value of the new model is 339.38 which is slightly worse than in the original model (337.72).

HR: Hazard ratio; CRP/Cl⁻: C-reactive protein-to-serum chloride ratio; MCI: modified Charlson index; PhA: phase angle; FM: fat mass; Kt/V (HW): Kt/V obtained by Hume-Weyer's formula; IDWG: interdialytic weight gain.

Figure S1: Kaplan-Meier estimate of all-cause survival probability by CRP and Serum Chloride levels.

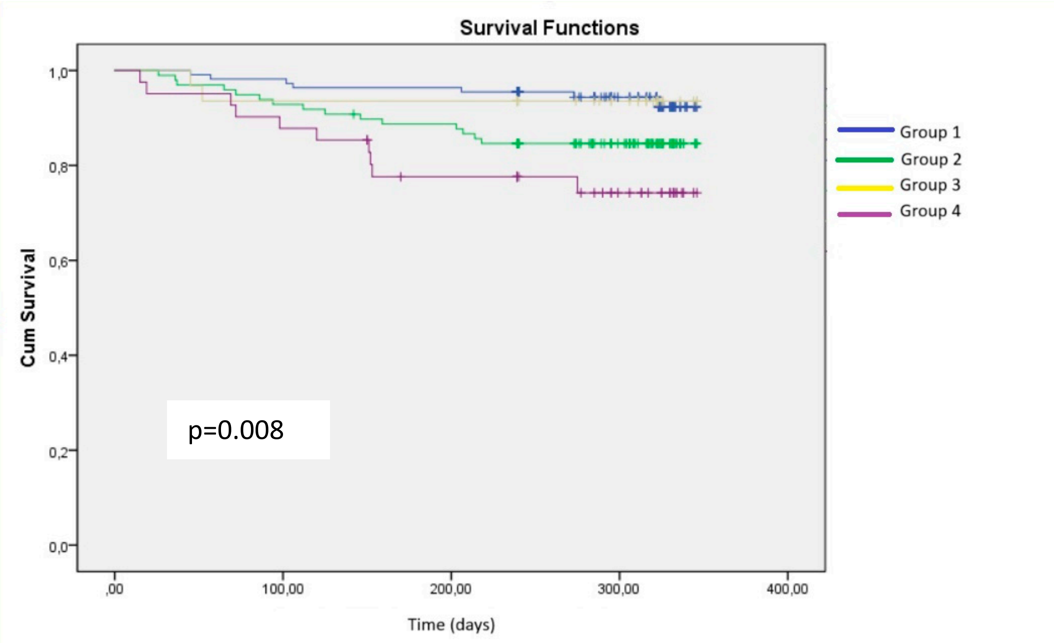

*Patients were stratified into four groups according to median CRP and hypochloremia. Group 1: No hypochloremia and lower CRP levels. Group 2: No hypochloremia and high CRP levels. Group 3: Hypochloremia and lower CRP levels. Group 4: Hypochloremia and High CRP levels. CRP: C-reactive protein.
